# Implantable hearing devices

**DOI:** 10.3205/cto000145

**Published:** 2017-12-18

**Authors:** Matthias Tisch

**Affiliations:** 1 Department of Otolaryngology, Head & Neck Surgery, Bundeswehrkrankenhaus Ulm, Germany

**Keywords:** hearing loss, hearing disorder, middle ear implant, active middle ear implant, fully implantable systems, partially implantable systems, bone conduction implants, passive systems, active percutaneous systems, active transcutaneous systems

## Abstract

Combined hearing loss is an essential indication for implantable hearing systems. Depending on the bone conduction threshold, various options are available. Patients with mild sensorineural deafness usually benefit from transcutaneous bone conduction implants (BCI), while percutaneous BCI systems are recommended also for moderate hearing loss. For combined hearing losses with moderate and high-grade cochlear hearing loss, active middle ear implants are recommended. For patients with incompatibilities or middle ear surgery, implants are a valuable and proven addition to the therapeutic options.

## 1 Introduction

**Figure 1:**
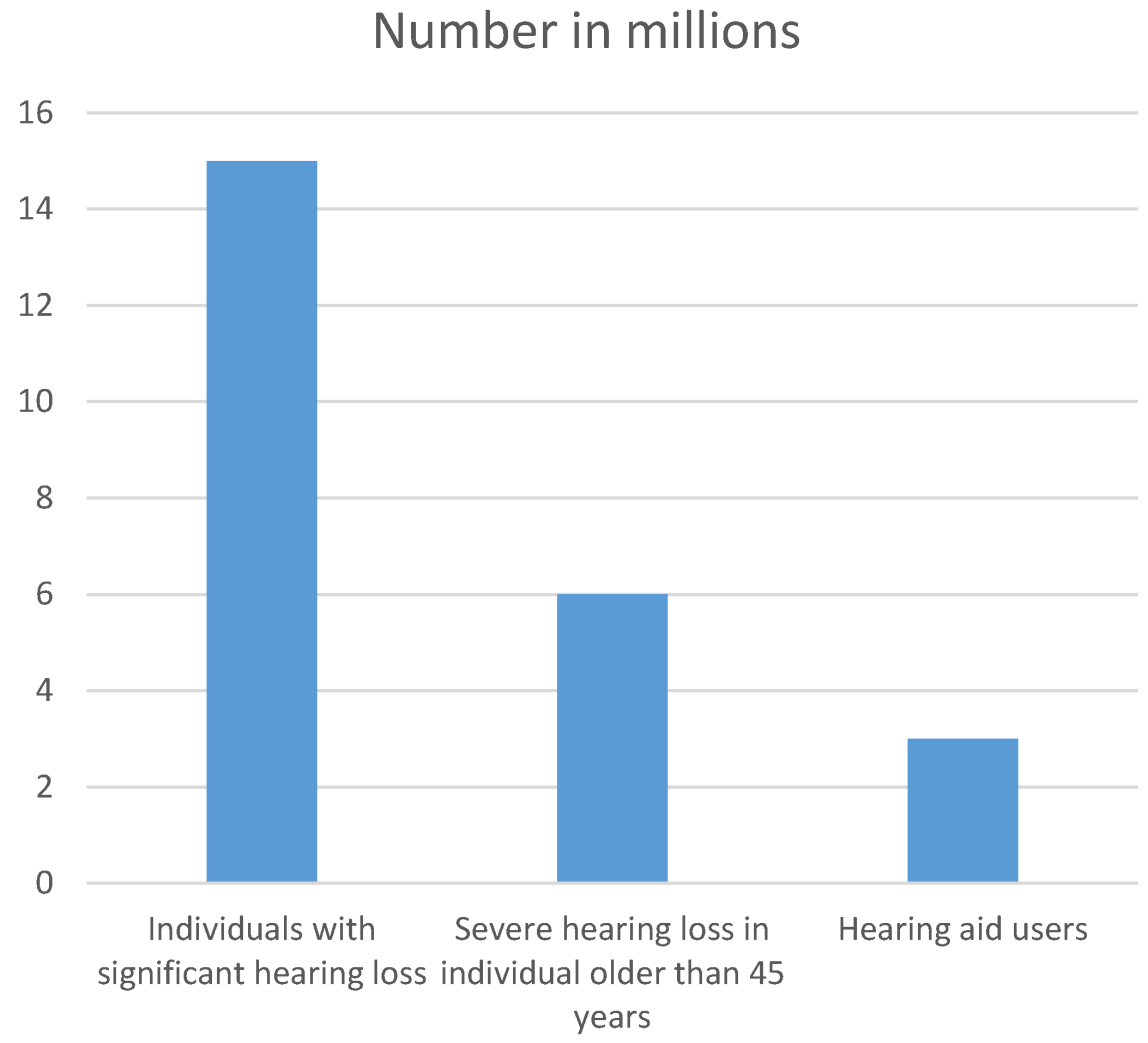
Number of people with hearing impairment and users of hearing aids in Germany in 2015 Source: Federal Statistical Office

According to data of the Federal Statistical Office, about 15 million people in Germany suffer from hearing loss, one of five uses a hearing aid (Figure 1 (Fig. 1)) [1]. Projections suggest that a significant percentage of those patients achieves an improved hearing in the speech audiometry due to the hearing aid, but has no real benefit according to the criteria of the Abbreviated Profile of Hearing Aid Benefit (APHAB) [2].

Because of the high complexity of the hearing process, still today the individual treatment of hearing impaired people is a great challenge. Especially in the context of moderate and high-grade hearing loss, conventional hearing aids reach their limits. Based on the S1 guideline entitled “Active implantable hearing systems for hearing losses” [3], implantable hearing systems are recommended for those patients.

This guideline that is currently in the revision process, the new version is expected to be issued in May 2017. It is generally valid for all patients who cannot be sufficiently supported with conventional hearing aids, whether this is due to medical or audiological reasons. The following indications are defined [3]:

- Mere sensory hearing disorder reaching the indication range of sound enhancing hearing aids
- Combined hearing losses in the context of functional disorders of the middle ear that cannot be treated otherwise by ear surgery with an expected benefit and that are not in the indication range of cochlea implantations
- Mere conductive hearing losses
- Malformations of the ear with one of the above-mentioned types of hearing disorder
- Incompatibilities of foreign bodies in the auditory canal (e.g. ear moulds)
- Chronic otitis externa or eczema of the auditory canal or chronic diseases of the outer ear

If the above-mentioned indication criteria appear in both ears, a bilateral treatment is indicated. 

The functional principle of implantable hearing systems consists of transforming the registered sound into electric signals and then into micromechanical vibrations that are transmitted to the ossicular chain or directly into the inner ear. Due to the clearly better amplification, implantable hearing systems do not only provide a better sound quality, but also a more differentiated speech recognition based on less distortion [4]. 

Also medical reasons such as intolerable occlusion and recurrent inflammations of the auditory canal, social reasons such as the job situation or the patient’s individual preferences may play a fundamental role when deciding in favor of an implantable hearing system. Often a successful social re-integration – job- or private life-related – is the actual clinical “bar” [4]. The implants have to fulfil the major expectations of possibly perfect hearing, high reliability, low failure rate, low surgical risk, reasonable expenses, and low visibility.

Initially, active middle ear implants were exclusively applied in patients who could not be satisfactorily rehabilitated with conventional hearing aids due to medical reasons. The development of the round window-vibroplasty technique allowed the additional treatment of patients with conductive and combined hearing losses [5], [6], [7]. Especially in the context of this indication, active middle ear implants have an advantage compared to conventional hearing aids and contribute to a higher quality of life regarding the hearing ability.

For decision making, the AWMF guideline recommends the individual discussion of diagnostics and indication in an interdisciplinary board consisting of ENT specialists, audiologists, and other disciplines (e.g. radiologists) [3]. The definitive decision, however, is up to the patient himself.

**Figure 2:**
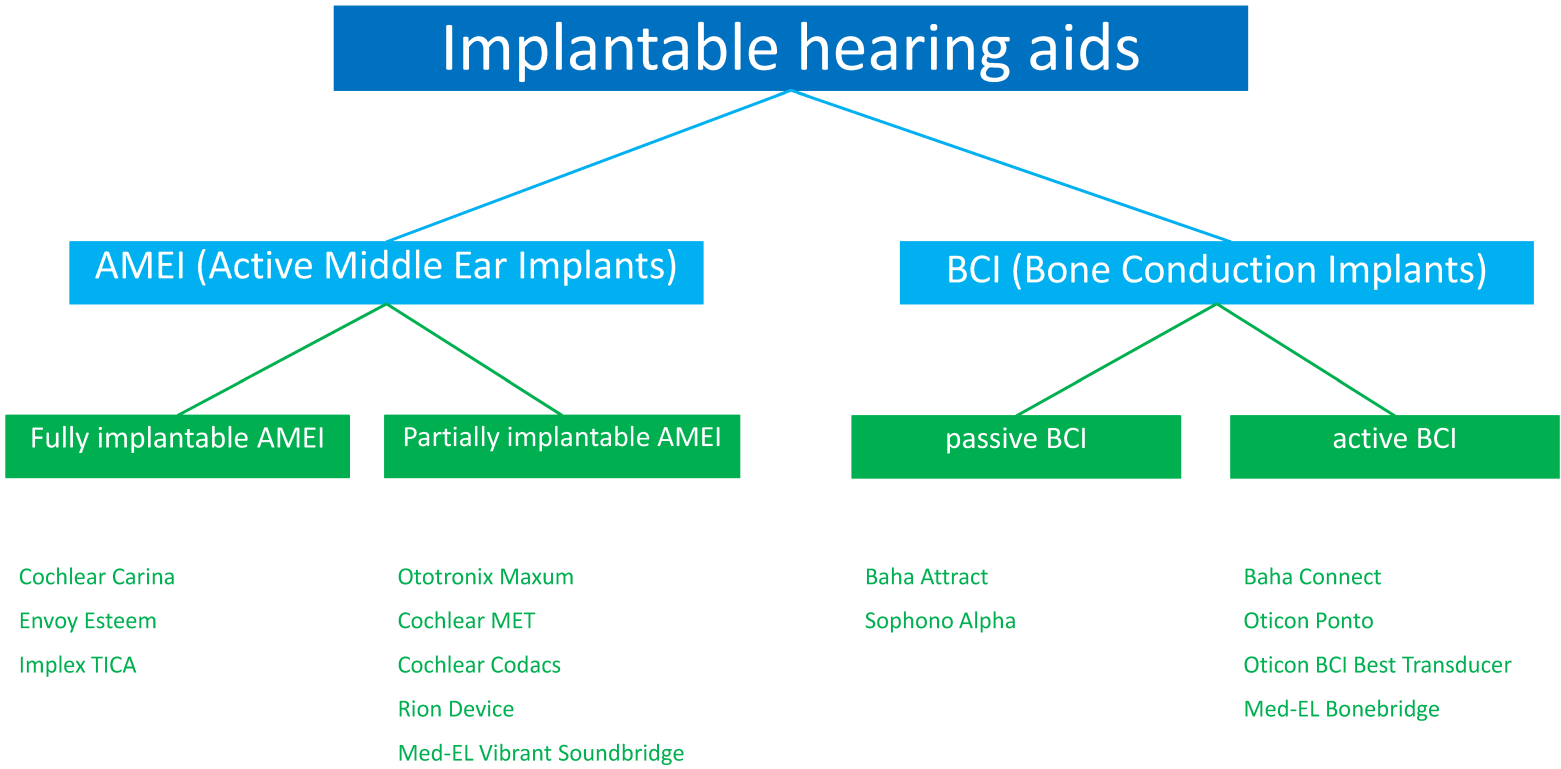
CE certified implantable hearing systems that are currently and were formerly approved in Europe

Implantable hearing systems are classified into partially and fully implantable hearing systems (Figure 2 (Fig. 2)). While for partially implantable models only parts of the hearing aid are implanted, all single components are implanted in the context of fully implantable hearing systems. Both systems have several advantages and disadvantages (Table 1 (Tab. 1)) [8] that led to the development of different systems.

## 2 Historical development

Meanwhile the history of middle ear implants counts more than 80 years. In 1935, Wilska implanted iron particles directly to the eardrum in order to stimulate the ossicles by means of a magnetic field [9]. In the 1950ies, 28,000 mA were necessary to generate a signal of 85 dB SPL; today the same performance is achieved with 3 mA.

### 2.1 Active middle ear implants (AMEI)

In 1959, Rutschman fixed 10 mg magnets at the malleus that was set into vibrations by an electromagnetic coil [10]; in 1973, the first mechanical implant was inserted by Frederickson et al. from the University of Washington, USA [11]. In 1977, finally the first series of partially implantable hearing system was developed, the “bone anchored hearing aid” (Baha). It was coupled to a fixing screw anchored in the cranial bone that transmitted vibrations via bone conduction to the inner ear and in this way bypassed the eardrum. With some modifications, the Baha and subsequent products are still manufactured today. They require a reduced surgical intervention without opening the middle ear and without manipulation of the ossicular chain.

Since the 1970ies, the research on magnetic fixation at the tympanic membrane was intensified. In 1973, the group around Goode and Glattke performed experiments with magnetic fixation at the malleus, the long incus process, and the oval window [12]. In 1988, Heide et al. positioned an induction coil in the outer hearing canal and a magnet at the ossicles in order to achieve an improved hearing. As a result, middle ear implants with piezoelectric transducers were developed [13]. 

In the 1990ies, different university institutions tested own implantable hearing systems, however, none of them became marketable nor was it produced in series. Maniglia et al. could show in cats that a magnet measuring 0.5–1.0 mm that was fixed at the incus, led to a hearing improvement of 22 dB [14]. In 1996, a trial was performed in patients with moderate to severe conductive hearing loss [15], however, it was not further pursued.

Another procedure with the objective to bypass the (intact) ossicular chain was investigated in Guinee pigs at the same time. For this purpose, the electromagnetic coil was implanted in the skin surface and the NdFeB magnet was fixed at the membrane of the round window. This prototype was never tested in humans. Also the attempts of Kartush and Tos to implant an electromagnetic coil in the outer auditory canal and a 30–45 mg magnet at the tympanic membrane, did not lead to success in the long term. The results of this method showed audiologically similar results as well-adjusted hearing aids [16], but a long-term study published in 2002 revealed that none of the patients used the system anymore [17].

Alternative, non-commercially approved AMEI with different transducers were developed among others at the University of Dresden (hydroacoustic transducer), the University of Bordeaux (piezoelectric transducer), and the University of Tübingen (piezoelectric implant at the round window with optic infrared) [18], [19], [20]. The earlens tympanic contact transducer was developed by Perkins and Shennib in 1993 based on a SmCo magnet that was implanted at the tympanic membrane in a soft silicone lens. In 1996, a study with 7 patients was published who had used the system for 3 months [21]. The load at the eardrum led to a loss of 5 dB, the functional gain, however, amounted to 25 dB. Further investigations in 2010 reached a threshold of 60 dB hearing loss [22]. Up to now, the system is not FDA approved.

The first fully implantable hearing system was the TICA LZ 3001 (“totally implantable cochlear amplifier”) of the Implex Company that came on the market in 1998. Since 1988, it had been developed in Tübingen by Leysieffer and Zenner. The system was specially conceived for moderate to high-grade sensory hearing loss and led to an improved word recognition and a significant functional hearing gain in several investigations compared to the preoperative, untreated situation.

In 1996, Ball developed the Vibrant Soundbridge (VSB), a system that was initially developed by Siemens Company to be produced in series (Symphonix Soundbridge). Later, Med-El Company included the system in their product range and developed the current function. The first implantation was performed in 1996 by Fish in a patient with mere sensory hearing loss. In August 2000, the Vibrant Soundbridge (VSB) was the first FDA approved AMEI for patients with sensorineural hearing loss [23].

In Europe, 3 companies with approved and certified systems are actively present in the market: Med-EL, Otologics, and Envoy Medical Corporation.

### 2.2 Bone conduction implants (BCI)

The idea to use the body’s ability to transmit sound via bone conduction, is rather old. Already in 1757, a doctoral thesis was published by Jorrissen on this topic, written in Latin. It was cited in a contribution to the *Journal de Physique* in 1783 about “hearing by means of the teeth and part of the hard palate”. In 1821, Itard developed the first bone conduction hearing system, which was a type of megaphone fixed at the patient’s teeth. Finally, in 1925, the first patent was granted for the “Bone Conduct Vibrator”.

Via the dentiphone for dentures users in 1939 and glasses with bone conduction function in the 1950ies, basic research was intensified in the 1960ies. The Swedish physician Dr. Branemark introduced the term of “osseointegration”, i.e. the integration of an implant into bony tissue.

The breakthrough came in the 1970ies: After the development of a tooth implant with bone conductive function [24], data of the first three patients with a bone-anchored hearing implant were presented in 1977. Since 1978, the system is distributed with the name of Baha (bone anchored hearing aid). After 5 years of long-term follow-up, all patients involved in the investigation were very satisfied, and no implant loss was registered. The first international workshop on bone-anchored implants took place in 1986, and in 2005 Cochlear Company acquired the Baha implants. It developed the first Baha with a digital audio-processor and directional microphone. In 2012, Med-EL launched the worldwide first active bone conduction implant. Currently, 4 international companies are working actively in the field of bone conduction implants: Cochlear, Oticon, Med-EL, Sophono.

## 3 Active middle ear implants (AMEI)

During the last 10 years, miniaturization and digitization led to significant improvements of conventional hearing aids and thus restricted the audiological spectrum of indications for AMEI. Beside medical (e.g. occlusion of the hearing meatus, recurrent inflammation) and esthetical reasons, the low tolerance of conventional hearing aids regarding humidity is a clearly defined field for the use of implantable hearing systems.

The amplified electric signals are transformed into mechanical vibrations and transmitted to the sound conduction apparatus of the middle ear (tympanic membrane and ossicles) or to the cochlea [25]. The necessary electromechanical transducer may work on an electromagnetic or piezoelectric basis (Table 2 (Tab. 2)) [26], [27]. In the context of electromagnetic transducers, the vibrations are generated between a current-supplied coil and a magnet [28], for piezoelectric transducers, they are generated by the current-induced relative change in the length of a piezoelectric crystal [29].

For the use in active middle ear implants, both systems have specific advantages and disadvantages. Electromagnetic transducers have a higher maximal output amplitude, but they consume more energy for the same acoustic performance. Furthermore, they are not MRI compatible. The piezoelectric transducers are less distorting [30], but their stiffness leads to a higher resistance of the sound conduction apparatus when they are coupled to the ossicles.

### 3.1 Fully implantable systems

#### 3.1.1 Cochlear Carina

**Figure 3:**
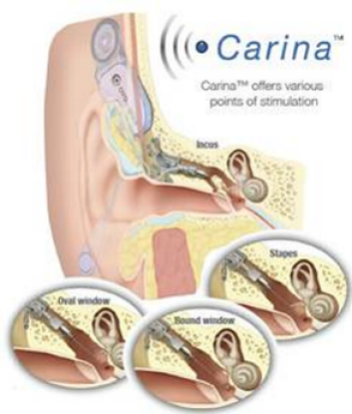
Structure of the Cochlear Carina system

The fully implantable system of Carina (Cochlear Company, Figure 3 (Fig. 3)) is based on the partially implantable system of the middle ear transducer (MET, see chapter 3.2.2). It works with an electromagnetic transducer that is connected with the ossicles. For this purpose, a coupling rod is inserted into a laser-surgically created mold of the incus [31], [32]. The transducer is fixed at the cortical edges of the mastoid cavity while a bony bed is created for the battery, the receiver coil, the signal processor, and the microphone in cranial or dorsal direction. Programming and charging of the battery are performed transcutaneously, the charging time amounts to about 1 hour for 16 hours operation time. Via a remote control device, the loudness of the Carina may be adjusted by the user and the device may be switched on and off.

**Advantages** are the missing occlusion effect, very good esthetic result (no stigmatization), excellent sound quality, good quality of life.

**Disadvantages** are the battery charging times and impaired MRI examinations.

**Indication:** The Carina implant is suitable for adults who suffer from moderate to high-grade sensory or combined hearing loss. The patients have to be at least 18 years old. As all electromagnetic transducers, it is not MRI-compatible.

**Surgical technique:** The Carina system is implanted via a postauricular incision (about 2 cm) and fixed at the ossicles [33]. Hereby, the mechanical load of the ossicles caused by the transducer can be intraoperatively monitored. By means of a micro-manipulator, the position of the coupling rod is optimized so that no mechanical displacement of the ossicular chain is caused. The bone bed for the electronic capsule and the microphone is created [34], [35]. High amplification can be achieved by fixing the implant with cortical screws [31], [36], [37].

There are 3 possible places to position the microphone:

- Anterior and above the external auditory meatus (temporal region)
- Posterior of the external auditory canal (retroauricular region)
- On the mastoid tip

The microphone is very sensitive to changes of the tissue thickness, movements may lead to feedback and undesired noise [35]. Currently, there is no consensus regarding the optimal position [34], it is recommended to find the best position intraoperatively by using the transducer loading assistant software of the manufacturer [38]. The tip of the transducer can be fixed on the long incus recess as well as the stapes arch or footplate, or at the round window [34], [38]. Tympanoplasty allows the implantation even in non-working ossicular structures and abnormal middle ear anatomy as long as the round window membrane is intact so that the transducer tip can be fixed [34], [39].

**Results:** In Europe, the system was approved after a clinical study with 8 patients [33], [38]. A phase-I-trial in the USA, revealed a slightly improved word recognition 3 months after implantation, however, 3 months later, the speech perception deteriorated because of device-related complications. After re-adjustment, the hearing performance was again improved [35]. The results of the current phase-II-trial are not yet published, but they seem to indicate very high differences regarding word recognition [40]. Similar observations have been made in smaller and larger studies (up to n=50) performed in different centers [39], [41], [42], [43].

The audiological results show consistently a good hearing gain for the Carina system even if long-term results are still not available. Compared to untreated patients, the middle functional gain amounted to 29 dB and 24 dB (p=0.0004) [34], [38]. The subjective patient satisfaction (measured by means of APHAB) revealed improvements in the areas of EC (49.8 vs. 19.9%), RV (57.7 vs. 44.8%), and AV (25.8 vs. 38.6%) (EC=Ease of communication; RV=Reverberation; AV=Aversiveness) [34].

However, in every published study, also problems with the implant are described. Beside feedback effects that may be met with re-adjustments, those problems include prolonged battery charging times, extrusion of the microphone cable up to complete communication loss of the implanted components and to complete failure of the implant [33], [35], [38].

**Discussion:** In many studies, problems with feedback were reported. Hereby, the position of the microphone under the skin seems to be a crucial factor. Movements of the head and neck may lead to skin displacement that is responsible for disturbing noise. The microphone should be placed in that way that the skin thickness above the microphone remains stable also when head and neck are moved. Also the quality of the intraoperative coupling between the transducer and the ossicles impacts the performance of the implant. The transducer loading assistant software provides intraoperative information on the mechanical load of the ossicles by the transducer and should be applied if possible. By optimizing the coupling, the mechanical displacement of the ossicular chain – and thus stiffening of the ligaments – can be avoided [35], [44].

The outcome that can be achieved with the Carina system is similar to the one with conventional hearing aids. The fact that many patients prefer implantable hearing systems has no audiological background but is mainly due to the subjectively better quality of life. Clinical long-term data are currently not available. Furthermore, data on the integrity of the incus after manipulation for insertion of the coupling rod are missing. In this context, alternatively possible couplings should be verified.

#### 3.1.2 Envoy Esteem

**Figure 4:**
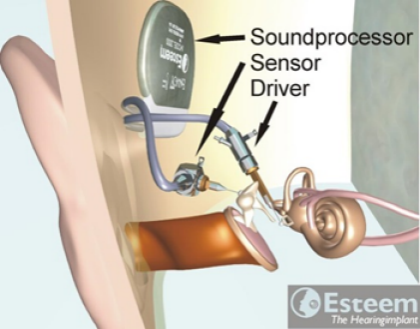
Structure of the Envoy Esteem system An animated presentation is found on https://www.youtube.com/watch?v=rRop0NSiKlM.

The Esteem system (Envoy Medical Company, Figure 4 (Fig. 4)) uses the natural sound immission at the tympanic membrane and does not need a separate microphone. The sound is registered by a piezoelectric transducer (sensor) at the incus and amplified; another piezoelectric transducer (driver) implanted at the stapes transmits the signal to the inner ear. The electric supply of the system occurs via special batteries that have a lifetime of about 3–9 years according to the manufacturer [45]; however, in some cases, the battery was already exhausted after 2 years. The battery has to be changed surgically.

**Advantages:** The signal registration occurs via the eardrum, no separate microphone is required.

**Disadvantages:** The ossicular chain has to be interrupted; long duration of surgery; surgical battery change.

**Indication:** Currently the device is only approved for adults with moderate to severe sensorineural hearing loss with a speech discrimination score of ≥40% under untreated conditions and normal anatomy and function of the Eustachian tube, middle ear, and tympanic membrane [45]. If the mastoid is suitable to insert the implant, has to be verified radiologically. Furthermore, the patients must have tested an optimally adjusted hearing aid for at least 30 days [46]. Contraindications for implantation might be post-adolescent and recurrent otitis and Menière’s disease.

**Surgical technique:** The 2 piezoelectric transducers are fixed in the mastoid cavity by means of hydroxyl apatite after retroauricular incision. In order to avoid feedback of the triggered stapes on the microphone placed at the incus, the ossicular chain has to be interrupted in the incudostapedial joint or at the long incus recess. Coupling to the ossicles is stabilized with bioglass cement. Attention must be paid to a sufficient distance between the incus and the sensor in order to have enough space for the natural movement of the eardrum and the (remaining) ossicular chain in cases of atmospheric pressure variations [47], [48].

**Results: **The results of a first phase-I study with 7 patients was sobering. 4 patients did not benefit from the system because of leaks of the cases; only after revision, 5 patients could be included in the postoperative examinations. The results after 2 months were better compared to the preoperative, untreated condition, but not better than optimal hearing aid fitting.

In phase II of the clinical investigations of 2004–2009, the speech recognition threshold was 12 dB higher compared to optimal hearing aid fitting, the average hearing gain amounted to 27 dB, the word recognition was improved by 22% [48]. The subjective perception of the quality of life was evaluated by means of a specifically developed questionnaire in a series of studies [48], [49], [50], in some aspects completing the Glasgow Benefit Inventory (GBI) score. In summary, slight improvements could be measured that were more significant in patients with moderate hearing loss compared to patients with severe hearing loss.

In 3 patients, secondary facial paresis occurred, 3 other patients had to undergo revision surgery because of insufficient audiological results [48]. Further undesired effects were wound complications requiring revision [47], [51] and disturbances of the chorda tympani [51].

**Discussion:** The clinical studies could confirm the desired effects of fully implantable hearing systems. Missing occlusion and low distortions lead to a subjectively better hearing perception than conventional hearing aids. One particularity of the Esteem implant is that the signal registration occurs via the tympanic membrane so that a microphone is not required.

However, some disadvantages must be mentioned. Those are among others complex surgeries with obligatory interruption of the ossicular chain to avoid feedback that may lead to additional iatrogenic conductive hearing loss in addition to the existing sensory hearing loss when the device is switched off. Furthermore, the battery can only be changed surgically. The lifetime of the battery mentioned by the manufacturer amounts to 9 years, in practice, however, it is not achieved.

### 3.2 Partially implantable systems

#### 3.2.1 Ototronix Maxum

**Figure 5:**
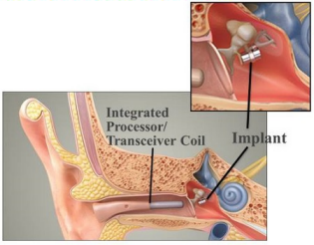
Structure of the Maxum system

The partially implantable Maxum system (Ototronix Company, Figure 5 (Fig. 5)) is based on a technology that was previously already known as Soundtec-Direct-Drive hearing system (Soundtec Company). The implantable component is the same in both devices. The Maxum system has a combination of digital sound processor and electromagnetic coil (integrated processor transceiver coil, IPC) in the auditory canal. The sound waves are received here and amplified. The coil generates an electromagnetic field that stimulates the magnet fixed at the incudostapedial joint [52].

The development started in the 1980ies at the House Ear Institute (USA). After initial difficulties – due to oxidation problems, the magnet had to be changed frequently – the phase I of the clinical studies started in 1998; in 2001, the system was the second FDA approved AMEI. Despite 600 implanted patients, it was withdrawn from the market in 2004 and the technology was taken over by Ototornix in 2009. One of the reasons was probably a rattling noise that was perceived also without audio-processor.

**Advantages:** Easy and rapid intervention that can also be performed under local anesthesia without any problem.

**Disadvantages:** The ossicular chain becomes heavier, often conductive hearing loss cannot be avoided, the ossicular chain has to be interrupted.

**Indication:** The Ototronix Maxum system is suitable for adults with moderate to high-grade sensory hearing loss. As all electromagnetic transducers, the Maxum is not MRI compatible [53], [54].

**Surgical technique:** The surgical technique can be compared to stapedoplasty and may generally be performed under local anesthesia. After incision and mobilizing the auditory canal flap, the tympanic membrane is tilted and the chorda tympani is neurolyzed. Then the stapes superstructure is exposed.

When the implant is inserted, attention must be paid that it is very delicate and must not be touched directly. The open part of the fixation sleeve is placed around the stapes head. It consists of nitinol and can be closed for example with the laser and thus fixed at the stapes head [52].

**Results:** In 2002, the results of the phase-II study with 103 patients were published [55]. They had a functional hearing gain of 7.9 dB and a better speech understanding of 5.3% compared to optimal fitting of hearing aids. In several studies, also the subjective patient satisfaction was assessed by means of questionnaires [55], [56]. They showed a statistically significantly higher satisfaction as well as a reduction of acoustic feedback and occlusion effects in comparison to conventional hearing aids. Postoperatively, an average of 1–3 session for fine tuning of the IPC were necessary [52]. 

Some undesired events were observed in the context of the approval study; those were earaches and a changed gustatory perception. In the context of subjective assessment of complaints by the patients, 55% reported about movements of the magnet that sometimes even required removing the implant [57].

**Discussion:** It is the case of a simple system that can be inserted rapidly, however, up to now it could not achieve a breakthrough. This is mainly due to the fact that the benefit compared to modern hearing aids is rather low and so the disadvantages strongly overbalance.

#### 3.2.2 Cochlear MET

**Figure 6:**
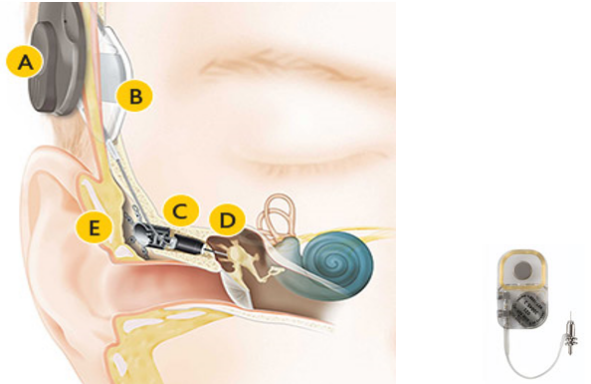
Structure of the MET system An animated presentation is found on https://www.youtube.com/watch?v=hBe1mFZCPcc.

The MET system (middle ear transducer, Cochlear Company, Figure 6 (Fig. 6)) is a partially implantable active middle ear implant that was meanwhile further developed to the Carina as fully implantable hearing system (see chapter 3.1.1). Both systems dispose of an electromagnetic transducer that is connected to the ossicular chain via a coupling rod. The external sound processor of the MET consists of a microphone, a digital sound processor, a magnet, and a coil [58]. Beside sensory hearing loss, the system can also be applied for conductive or combined hearing losses with a modified coupling rod. Even in the context of ear deformities [59] and otosclerosis surgery [60] it has already been used.

**Advantages:** Very efficient system, higher amplification than VSB.

**Disadvantages:** Very complex surgical intervention, coupling at the incus with disturbance of the integrity of the incus (risk of necrosis).

**Indications:** The Cochlear MET system can be used in adults with moderate to high-grade sensory hearing loss or moderate to high-grade combined hearing loss. The hearing threshold should not be lower than 65 dB HL in the frequencies of 250–550 Hz and 80 dB as of 1000 Hz and be stable. Furthermore, anatomical limitations for the approach to the mastoid antrum must be observed [61]. Contraindications are retrocochlear and central hearing disorders, persisting otitis media, or severe concomitant diseases. Before MET implantation, a hearing aid should be tested at least for 3 months. The MET implant system is not MRI compatible.

**Surgical technique:** The surgical technique corresponds to an extended antrotomy, however, with complete exposition of the incus. After insertion of the implant, a mold is created in the incus by means of laser to insert the coupling rod that transmits the vibrations to the ossicular chain.

**Results:** An analysis of 282 patients from more than 100 centers assessed the functional hearing gain, speech recognition, and subjective perception before implantation as well as 2, 3, 6, and 12 months afterwards [62]. Speech understanding and subjective assessment of the patients were postoperatively improved. The amplification for the frequencies of 250–6000 Hz showed a clear peak of +38 dB at 1500 Hz and still 27 dB at 6000 Hz [63].

**Discussion:** The minimal change of the air conduction threshold after implantation and unchanged bone conduction threshold is explained by the mass load of the coupling [64]. In the context of a study from the Netherlands, the patients achieved a better speech discrimination with the MET as of a sensory hearing loss of 65 dB compared to conventional hearing aids and the Vibrant Soundbridge (VSB) [65].

#### 3.2.3 Cochlear Codacs

**Figure 7:**
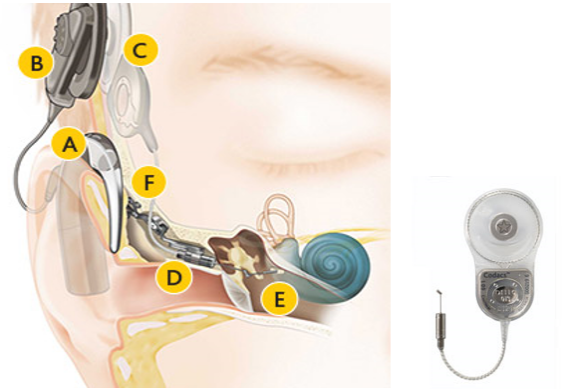
Structure of the Codacs system An animated presentation is found on https://www.youtube.com/watch?v=VKIH-IAkxS8.

The Codacs system (Cochlear Company, Figure 7 (Fig. 7)) consists of an external programmable speech processor and the implant itself that is fixed at the skull. By means of a coupling rod, at the end of which an artificial incus is fixed, the vibrations are transmitted to the perilymph over the stapes footplate [66]. In cases of maximal output power, theoretically an amplification of up to 94 dB is possible (manufacturer’s data).

**Advantages:** High amplification, natural hearing impression in comparison to CI.

**Disadvantages: **Complex surgical procedure, with high risks for the inner ear.

**Indications:** The Codacs system is indicated for high-grade hearing loss or combined hearing loss close to deafness with sufficient cochlear reserve. The minimal bone conduction threshold is 75 dB at 500 Hz and 90 dB at 2 kHz. For patients with non-ventilated middle ear and missing posterior wall of the auditory canal, proceeding in several steps is recommended (subtotal petrosectomy, closure of the external auditory canal, obliteration of middle ear and mastoid) [66].

**Surgical technique:** In patients with intact posterior wall of the auditory canal and ventilated middle ear, the soft parts are lifted from the mastoid after retroauricular incision; a periostal pouch is created in occipital direction for later insertion of the implant with the transmission coil. After mastoidectomy and posterior tympanostomy, the ossicular chain is exposed. Then, a bone mold is drilled at the posterior lower edge of the mastoid and the fixation system is fastened with micro-screws. After insertion of the implant, the transducer is positioned in the fixation system over the stapes footplate. The stapes superstructure is removed and the footplate is opened, in analogy to stapedoplasty.

For patients with missing posterior wall of the auditory meatus or non-ventilated middle ear, subtotal petrosectomy is recommended with closure of the external auditory canal and the middle ear as well as the mastoid with belly fat. After lifting the belly fat, the implantation may be performed. 

**Results:** A first small study of patients with high-grade combined hearing loss and otosclerosis, showed substantial improvements of the hearing threshold, speech understanding, and subjective hearing perception. The speech understanding could be increased from initially 40% to postoperatively 100% (at 75 dB) [67]. Also a larger study from 2014 including patients with high-grade combined hearing loss showed significant differences [66]: in the free field the average improvement of the hearing threshold amounted to 50±9 dB vs. 38±11 dB for conventional hearing aids, the speech understanding increased from 25% to statistically significant 85% (at 65 dB). Even the subjective assessment by the patients was better in comparison to conventional hearing aids.

**Discussion:** The studies describe a certain variability of the results – similar to patients with advanced otosclerosis who received conventional stapedoplasty. The amplification of the Codacs is very high, and patients describe the hearing perception as natural. Compared to other systems that use mechanical elements for coupling to the ossicular chain and that are influenced by the mechanical properties of the residual middle ear structures, the direct coupling of the Codacs system to the perilymph shows very consistent results with low variance. Nonetheless, the surgical procedure is complex, and the prediction of the hearing outcome after implantation remains difficult [68], also because it may be a problem to determine the cochlear reserve in patients with bilateral high-grade hearing loss. The insertion of a Codacs system is generally also possible in patients with mere sensory hearing loss. But with regard to the low number of cases, far-reaching statements cannot be made.

#### 3.2.4 Rion Device

**Figure 8:**
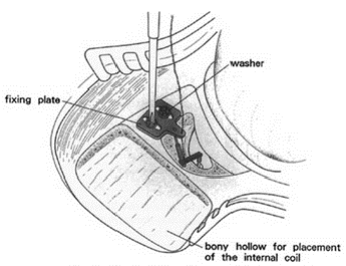
Structure of the Rion Device (according to [70])

Already in 1978, the Universities of Ehime and Teikyo in Japan started developing a partially and fully implantable hearing system with a piezoelectric transducer (Figure 8 (Fig. 8)) that was clinically tested by Rion since 1984 after successful animal experiments. In 1993, the system was approved for Japan [69]. In 2005, the company disappeared from the market because of missing economic efficiency.

**Advantages:** Good amplification, high patient satisfaction.

**Disadvantages:** Often conductive hearing loss cannot be avoided. Interventions at the mastoid and middle ear are necessary.

**Indications: **The Rion device was approved for patients with combined hearing loss due to severe middle ear damage that could not be surgically rehabilitated. 

**Surgical technique:** Opening of the ear from posterior and opening of the mastoid. Exposition of the stapes superstructure. Insertion and fixation of a fixation plate in the mastoid, insertion of the piezoelectric element and coupling to the superstructure, fixation with cement if needed. In cases of missing superstructure, also a middle ear prosthesis can be used to create a bridge between the coupling element and the footplate.

**Results:** From 28 patients who received a device of the first generation until 1989, 27 patients observed a significant hearing improvement [70]. However, in 17 patients also complications were documented: 8 patients developed significant tube ventilation problems, retraction pouches of the eardrum and/or cholesteatomas. In 4 patients, the system had to be explanted because of wound healing disturbances and fistula development. Other 4 patients suffered from progredient sensory hearing loss and had to be explanted [70].

In 1990, the second generation of the system was presented. The improvements were a thinner internal coil, a robust conducting wire, and a better amplification of 10 dB on average. Among 11 patients, 1 patient developed a retroauricular fistula and had to be explanted, 2 other patients developed a cholesteatoma. The functional hearing gain in both studies amounted to 36 dB on average with high patient satisfaction due to reduced feedback and natural sound in comparison to hearing aids [71], [72]. The mean useful life of the device was 16.6 years, the longest duration was 21 years.

**Discussion:** Despite the significant surgical efforts and the low number of cases, the results were good to excellent in the first as well as in the second series. In comparison to the TICA, however, the economic base was not considerable so that the system was not widely distributed and finally stopped.

#### 3.2.5 Implex TICA

**Figure 9:**
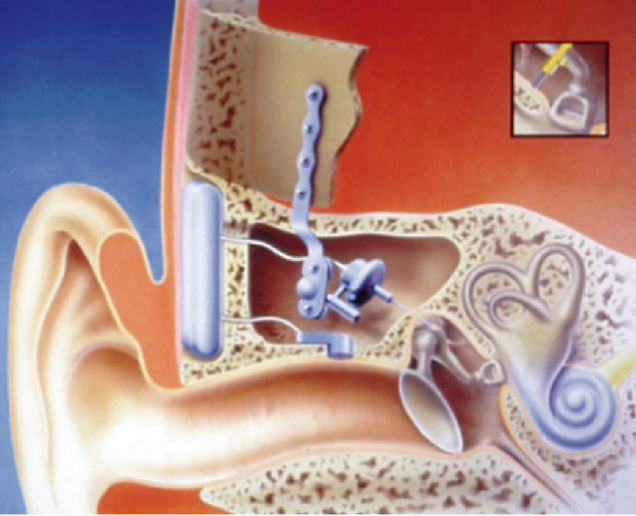
Structure of the Implex TICA

The hearing system TICA LZ 3001 (totally integrated cochlea amplifier, Implex Company, Figure 9 (Fig. 9)) was developed in Tübingen in the 1990ies. It consisted of 3 implantable modules: the processor unit with battery and digital audio-processor, the membrane receptor as microphone, and the piezoelectric transducer. All components could be completely implanted in the middle ear, the mastoid cavity (after mastoidectomy) and in the bone area behind the auricle. During the development of the single components, especially the safe implantability, the high quality of transmission (low distortions, high spectrum), biostability of the implanted components as well as high robustness against mechanical and electromagnetic influences were in the focus [73], [74], [75], [76]. In 1998, the TICA was the first fully implantable AMEI that received the approval for Europe. After insolvency of the Implex Company, the technology was taken over by Cochlear.

**Advantages:** Microphone in the auditory canal, mostly natural hearing, good rehabilitation in cases of steeply sloping high frequency losses.

**Disadvantages:** Short battery lifetime, complex surgical procedure.

**Indications:** Patients with moderate to severe sensorineural hearing loss [77].

**Surgical technique:** After mastoidectomy, the incus is exposed. Now the posterior wall of the auditory canal is exposed and thinned out. When drilling a mold to insert the microphone, special attention must be paid to preserve the integrity of the skin of the auditory canal. A support plate is fixed to which later the actor is attached, insertion and fixation of the hearing device in the area of the calvaria. Transmission can either be realized by means of an elbow clip, incus coupling, or coupling of a middle ear prosthesis.

**Results:** The published results are limited to a phase-III study with 20 patients from 2004. In a 6 months follow-up, 17 of 20 patients showed a functional hearing gain and better speech recognition.

##### Personal experience with the TICA system

The author treated 2 patients with the TICA system for steeply sloping high frequency loss. Both patients wanted to regain their hearing abilities without being stigmatized by a visible hearing aid and were highly satisfied with the amplification and the comfort of the TICA. The only limiting factor for both users was the lifetime of the battery. One of the patients was a general practitioner who was able to normally auscultate due to the optimal microphone of the TICA. Finally, the TICA hearing system was ahead of the times – the market was not yet ready for this new type of hearing aids. After some years when the Implex Company had disappeared and no support was available, both patients had to “switch” to partially implantable hearing systems of the VSB type.

#### 3.2.6 Med-EL Vibrant Soundbridge

**Figure 10:**
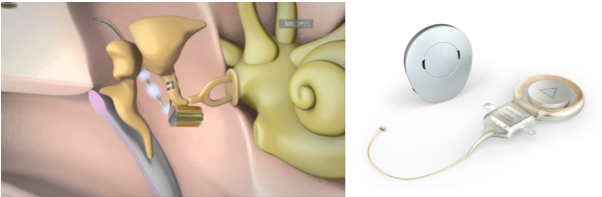
Structure of the VSB system An animated presentation is found on https://www.youtube.com/watch?v=gzpNY6Rk-Hc.

The Vibrant Soundbridge (VSB, Med-EL Company, Figure 10 (Fig. 10)) is currently the mostly implanted hearing system worldwide [78]. It had been developed by Symphonix Devices and was implanted for the first time in 1996. It can be used for several types of hearing impairment [79], [80]. In the external audio-processor, the microphone, transmitting coil, and battery are integrated, in the implant, there are the receiver coil with the processor element (vibrant ossicular prosthesis, VORP) and the electromagnetic vibrator (floating mass transducer, FMT) [81].

In the FMT, the sound signal is transformed into micro-mechanical vibrations due to the reaction force of the magnet moved in the coil. The FMT is either coupled to the ossicles, a middle ear prosthesis, or the round window and transmits the vibrations to the inner ear.

Since the FMT does not need to be anchored in the temporal bone, several coupling possibilities exist and the indication spectrum is large. Due to the anchoring in only one structure of the middle ear, the VSB is independent from the growth of the surrounding bone and even children may be implanted. Via a stimulation system (direct drive stimulator, DDS) the VSB can be brought into contact with the tympanic membrane before surgery in order to give a rough impression of the probable results. The processor technique of the VSB meanwhile contains programs for different hearing situations that the patient may select himself. With more than 50 publications, the VSB is the best investigated AMEI [74].

**Advantages:** The VSB is MRI compatible up to 1.5 Tesla and may be implanted also in children due to the single-point fixation. Variable coupling options allow the application in various pathologies.

**Disadvantages:** Acoustic shadow in the context of MRI examination of the skull.

**Figure 11:**
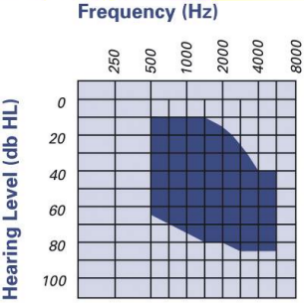
Audiological indication range for the Vibrant Soundbridge

**Indications: **The VSB is approved for adults and children from the age of 5 years with conductive and combined hearing losses (Figure 11 (Fig. 11)).

**Surgical technique:** There are different techniques for surgery and coupling. In the context of classic vibroplasty, the VORP is positioned in an appropriately drilled bone bed and the FMT is fixed at the long incus process. A conduction wire connects both elements [78].

**Figure 12:**
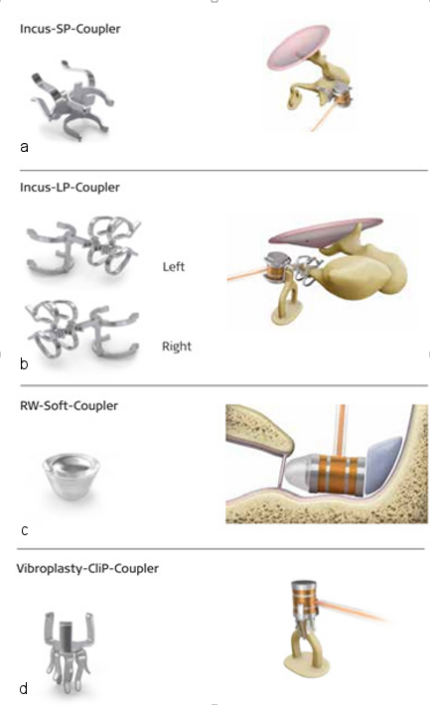
Different couplers of the Vibrant Soundbridge for positioning of the FMT at the short (a) or long (b) incus process, at the round window (c) or at the stapes head (d)

Because of the development of different couplers (Figure 12 (Fig. 12)), there are meanwhile many variations of this model [82], [83], [84]. In cases of irreparable deformity or damage of the outer and middle ear structures, the FMT may also be attached directly at the round window. In this way, conductive and combined hearing losses can be treated [85].

**Results:** Regarding the VSB, extensive studies have been published that had partly longer follow-up intervals and were evaluated in several meta-analyses [86], [87], [88]. Nine studies with a total of 153 patients compared the Soundbridge with conventional hearing aids. 6 of those 9 studies (n=129 patients) found a clinically significant benefit (>5 dB difference in the amplification) for the VSB. Regarding the speech recognition, the results vary, in some studies data on the statistical significance of the outcome are missing. A detailed investigation about the quality of life (n=51) declared a significant advantage of the VSB over conventional hearing aids in all evaluated subcategories (p<0.001) [89].

In comparison to the untreated situation – 29 studies with a total of 796 patients – the differences were higher and all studies confirmed a significant hearing gain of 27.1 dB on the average for the VSB. The Glasgow Benefit Inventory (GBI) as indicator for the quality of life, was used in 4 studies. While the VSB was rated better in the subcategory “general” in all 4 studies, the subcategory “physical” obtained an improvement only in 1 study, in 2 studies the results were comparable, and in 1 the results deteriorated with the VSB [88].

**Discussion:** The VSB is suitable for different types of hearing loss and is also approved for children [90]. It is implanted in cases of sensory hearing loss when the fitting of conventional hearing aids is not possible because of chronic otitis externa or incompatibilities, as well as in cases of conductive or combined hearing impairment when conventional tympano- and ossiculoplasty cannot improve the hearing ability. Since the VSB is not fixed at the skull bone, a relevant energy loss is observed in low frequencies. Patients with hearing losses of 250–500 Hz need a higher loudness that is limited due to energy reasons [91].

Some of the studies show an important variance regarding the functional hearing gain and speech recognition. This may be due to the high variability of the FMT coupling – with different effectiveness – as well as to other co-factors that are not assessed in the context of the studies (e.g. pathology, time and severity of hearing loss). The differences regarding the speech recognition (untreated: 0–72%; with VSB: 55–95%) have methodical origins because many recognition systems were applied.

Since the VSB provision describes a relevant hearing gain in all clinical studies compared to the untreated situation, the VSB can be considered as effective therapeutic option. Better speech understanding vs. hearing in quiet could only be shown significantly in one study [92]. In noise, the patients with VSB achieved more often better speech understanding than with conventional hearing aids. A long-term study revealed stable results over the follow-up time of 5–8 years [93].

A consensus statement of 2014 on the implantation of the FMT at the round window [94] describes the technique as reliable procedure for patients with conductive and combined hearing loss. Similar results for this procedure were described in a retrospective analysis of surgical and audiological data of 21 patients between 19 and 62 years [95] and in a long-term study (follow-up of 12–71 months) in children and adults [96].

## 4 Bone conduction implants (BCI)

Bone-anchored hearing systems (bone conduction implants; BCI) are mainly applied in patients with conductive hearing loss whose inner ear function is mostly intact. Since the sound energy is transmitted via bone conduction, the amplification of the inner ear is limited. Nonetheless, a similar functional hearing gain can be achieved in mere conductive hearing loss compared to active implantable systems. The most important advantage of BCI over AMEI is the clearly reduced surgical effort – without opening the middle ear or manipulating the ossicular chain. This fact leads to a low complication rate.

In the group of BCI, the difference must be made between passive and active options, the active BCIs are classified into percutaneous and transcutaneous systems (Table 3 (Tab. 3)).

### 4.1 Passive BCI

#### 4.1.1 Baha Attract

**Figure 13:**
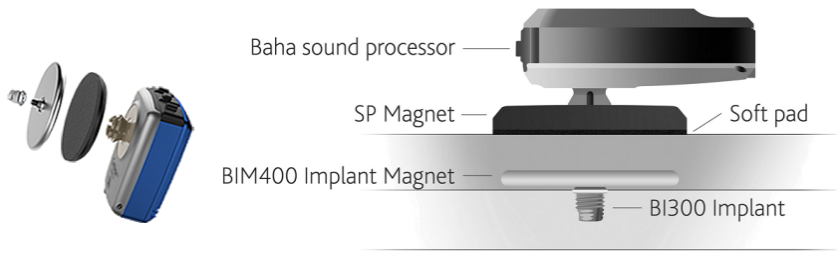
Structure of the Baha Attract

The Baha (bone anchored hearing aid, Cochlear Company, Figure 13 (Fig. 13)) is clinically used for nearly 40 years now. Since 2014, also the transcutaneous system of Baha Attract is applied beside the classical percutaneous system (Baha Connect, see chapter 4.2.1.1). The information is transmitted through the closed skin and the sound processor is kept in place by a magnet over the implant screw, which reduces possible wound problems. According to the sound processor, the maximum amplification amounts to 40–60 dB between 250 and 4000 Hz [97], [98], [99].

**Advantages: **MRI compatible up to 1.5 T, good skin tolerance.

**Disadvantages:** High contact pressure, the amplification is reduced by the skin thickness.

**Indications:** The Baha system can be applied for treatment of conductive hearing loss, combined hearing loss, and unilateral sensorineural deafness in adults and children from the age of 5 years.

**Surgical technique:** Usually, the intervention is performed under general anesthesia. After retroauricular incision, identification of the implantation site. Insertion of the screw (if it is not a system change) and fixation of the magnet plate, afterwards closure of the wound. After 6 weeks, the audio-processor is activated and fitting is performed.

**Results: **First experiences in 12 patients showed a statistically significant hearing improvement with an average threshold of 56 dB without and 37 dB with Baha hearing system [100]. Also the subjective patient evaluation in the APHAB was significantly better in the subcategories of reverberation (p=0.016), of background noise (p=0.035), and of global score (p=0.038). In the parallel assessed pain score, exclusively low values were documented, an initial sensation of deafness was regredient in most of the patients.

The signal loss due to transcutaneous transmission (about 5 dB at 1000 Hz and 20–25 dB at more than 6000 Hz) can be partly compensated by the individual adjustment of the sound processor, especially in the range of 3000 Hz [101]. Another study with 27 patients confirmed the hearing improvement and emphasized the easy implantation and the complication-free healing. At the end of the 9 months follow-up time, all patients still used their implant and rated it as positive [97]. Also a recent multicenter trial from 2016, showed a significant improvement of the speech understanding after percutaneous implantation of Baha in patients with bilateral combined hearing loss, bilateral conductive hearing loss and single-sided deafness [102].

In the mentioned studies, only 3 severe complications were registered in 128 patients that required revision (2.3%). All of them had skin-associated origins.

**Discussion:** The Baha attract is a useful device to avoid screw extrusions that occur every now and again. The disadvantages are the rigid plate that may complicate the surgical intervention and the high contact pressure. The fact that many patients equipped with a Baha Connect can switch to another system without any problem is one of the great advantages.

#### 4.1.2 Sophono Alpha

**Figure 14:**
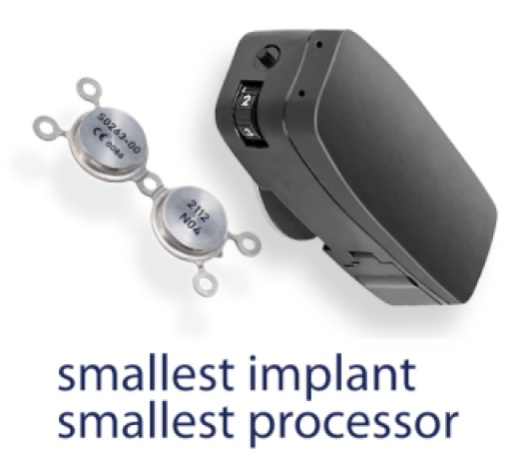
Structure of the Sophono Alpha

Also the Sophono Alpha system (Medtronic Company, Figure 14 (Fig. 14)) is a passive transcutaneous system. Currently, the model Alpha 2 is on the market consisting of a titanium implant and an external processor. As mount, a double magnet is fixed at the cranial bone [99], [103]. The particularly compact structure is supposed to ideally hide the processor completely in the hairline. The system is controlled by a digital 4-channel processor with 16 frequency bands.

**Advantages:** MRI compatible up to 3 T, good skin tolerance.

**Disadvantages:** low amplification (45 dB are not achieved), big external component.

**Indications:** The system can be applied for treatment of conductive hearing loss, combined hearing impairment, and unilateral sensorineural deafness in adults and children from the age of 5 years [103].

**Surgical technique:** The Sophono Alpha System (SAS) consists of 2 elements, an implantable magnet and the external audio-processor. Surgery is performed under general anesthesia. After identification of the correct position, the implant bed is created and the implant is inserted. The implant is fixed with titanium screws. After 4 weeks, the audio-processor may be activated and adjusted.

**Results:** The currently available study data mainly come from the model Alpha 1. In cases of conductive or combined hearing loss, the patients could not achieve understanding of monosyllables in the free-field without hearing system (average value ±10%), with hearing system they achieved an average of 86±17%. The mean audiometrically measured hearing gain in the free-field was 38±8 dB [104]. Patients with bilateral conductive hearing loss achieved a higher functional hearing gain than patients with combined hearing loss (21.9±10.4 dB vs. 6.2±5.3 dB) [105].

In one study, nearly every third patient (35.7%) had light to moderate skin problems [9]. Pressure sensation was reported in the context of positioning of the magnet plates. Hereby, it is recommended to start initially with low magnet strengths [104].

**Discussion: **Up to now, only very limited data are available. The comparably low maximum output levels restrict the application to cochlear hearing losses of <20 dB HL [106]. In comparison to systems established in Germany, no advantages can be seen that make a relevantly higher distribution more probable.

### 4.2 Active BCI

#### 4.2.1 Active percutaneous systems

##### 4.2.1.1 Baha Connect

**Figure 15:**
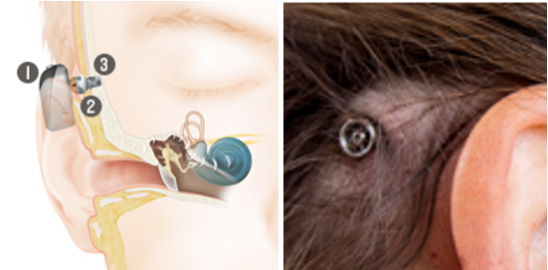
Structure of the Baha Connect, implant behind the ear

Since 1977, the percutaneous Baha Connect system (Cochlear Company, Figure 15 (Fig. 15)) is on the market. An abutment anchored in the bone transmits the sound in form of vibrations from the sound processor to the implant and then further through the bone to the inner ear. The sound processor can be adjusted independently from the system in cases of technical innovations (Baha 3 → Baha 4 → Baha 5). As all percutaneous system, the Baha Connect requires daily care.

**Advantages:** MRI compatible.

**Disadvantages: **Possible screw extrusion, intensive care is necessary.

**Indications:** The system can be applied for treatment of conductive hearing loss, combined hearing impairment, and unilateral sensorineural deafness in adults and children from the age of 5 years [107], [108], [109], [110], [111], [112], [113].

**Surgical technique:** The surgical technique is comparably simple and can be performed as routine intervention under local anesthesia. Thinning out of the skin is no longer necessary and is not recommended.

**Results:** Since the Baha Connect is already on the market for a long time, relatively many data are available. An early study from the 1990ies with 120 patients confirmed a functional hearing improvement of 29.4 dB on the average as well as an improved speech recognition of 41.6% for the model HC 200 of that time [114]. Meanwhile different processor models (single-channel and multi-channel systems) are used and were compared in numerous studies – such as the linear single-channel system Intenso and the non-linear multi-channel system BP110 [115], [116], [117]. Both systems improved speech recognition compared to untreated conditions, in quiet no difference could be identified between both sound processors. In cases of loud background noise, the speech understanding with the complex multi-channel system BP110 was better. In a study comparing this system with a transcutaneous Baha system, the authors found a better functional hearing gain for the percutaneous variant, however, the difference was not statistically significant [118].

Local skin reactions were the most undesired side effects, among them skin growths, infections, and necroses [118]. In 8.3% of the cases, those findings led to a loss of the implant over a follow-up time of 10 years, particularly in children [119]. The authors explain this observation by sports and playing activities that are associated with an increased risk of injury.

**Discussion: **Whereas many studies with follow-up intervals of several weeks or months were very promising, the results of a long-term study with an average follow-up of 7 years were rather sobering. Only in 56.6%, the system was still present and functional. The main reason for the abandonment (after an average of 5 years of use) were excessive background noise and insufficient improvements [120]. The authors emphasize the importance of continuous follow-up since the compliance can be clearly increased.

Regarding the surgical technique, the maximal thinning out of the skin was considered as most important measure for a long time to avoid reactions at the skin opening at the long term. Meanwhile it is considered as evident that a hydroxyl apatite coating reduces the bacterial development [106], [121]. The lesser invasive procedure has a positive effect on the surgery time and leads to esthetically better results [122].

According to current experience, the technique can also be applied in children [123]. Skin reactions in the area of the implant occur as frequently in children as in adults. Because of bone growth, the peri-implant bone may grow upward, which makes revision necessary in 10–30% of the cases [124], [125].

##### 4.2.1.2 Oticon Ponto

**Figure 16:**
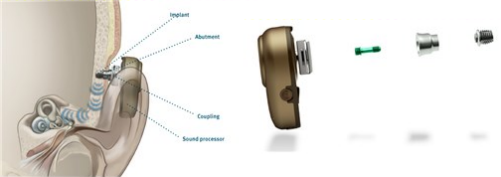
Structure of the Oticon Ponto

The Ponto system (Oticon Medical Company, Figure 16 (Fig. 16)) is also a percutaneous BCI system that comprises different processors (Ponto, Ponto Pro, Ponto Pro Power). A system consists of an external sound processor, the anchoring in different lengths (depending on the skin thickness), and the implant in the skull bone [126]. According to the manufacturer’s description, the conductive hearing threshold for Ponto and Ponto Pro amounts to more than 45 dB (at 500–3000 Hz) .

**Advantages:** MRI compatible to 3 T.

**Disadvantages:** Intensive care, screw extrusions.

**Indication:** The system can be applied for treatment of conductive hearing loss, combined hearing impairment, and unilateral sensorineural deafness in adults and children from the age of 5 years [126]. Patients with combined hearing loss should have an average bone conduction threshold of 55 dB HL or better.

**Surgical technique: **The surgical technique is comparably simple and can be performed routinely under local anesthesia. Thinning out of the skin is no longer required and is not recommended. After identification of the correct position, the screw is inserted; after osseo-integration, the audio-processor is activated and fitted.

**Results:** A crossover study compared Ponto and Ponto Pro Baha Connect BP100. The participants used the devices for a time of 25 to 63 days; among other parameters, speech recognition in loud noise was measured. The users’ satisfaction was assessed by means of questionnaires according to NSH and GHABP. At the end of the test, 8 of 12 patients decided for the Ponto Pro and 4 for the BP100. In terms of handling, visual appearance, and speech understanding, the Ponto Pro was superior. Regarding the “speech-in-noise” test, the Ponto Pro microphones showed better results with their directional feature [127].

The undesired side effects that were most frequently reported were skin associated, most of them unimportant. In 3.9% of all patients from 11 studies, revision was necessary.

**Discussion:** First short-time data seem to reveal a reliable implant function without undesired side effects. Only clinically less relevant skin reactions (Holgers grade 1) were frequently observed. A prospective trial with a longer follow-up time should provide information about the long-term benefit.

#### 4.2.2 Active transcutaneous systems

##### 4.2.2.1 Oticon BCI Best Transducer

**Figure 17:**
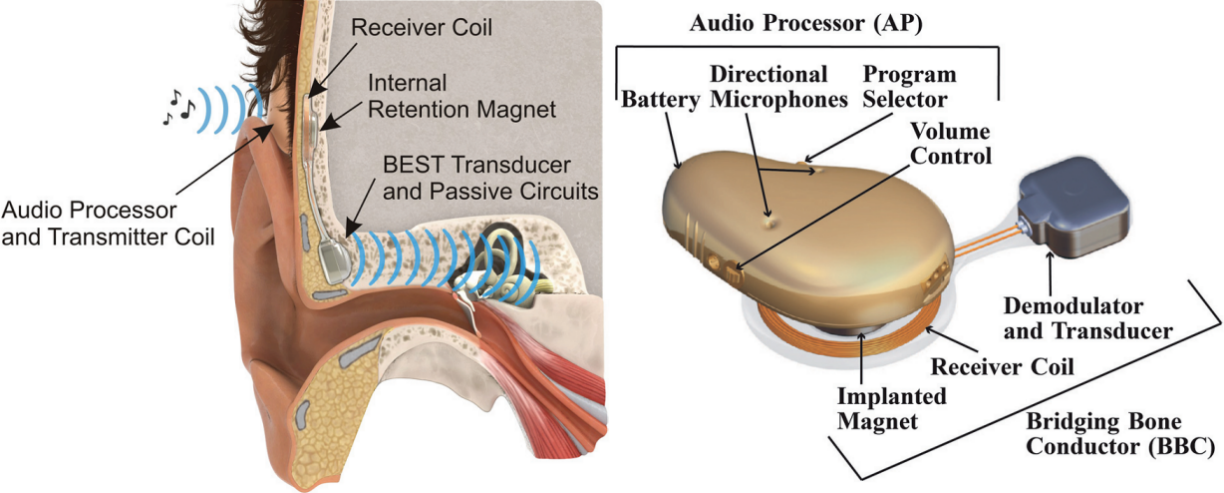
Structure of the Oticon BCI Best

The BCI Best Transducer (Oticon Company, Figure 17 (Fig. 17)) consists of an external audio-processor and the implant called Bridging Bone Conductor, a magnet, the receiver coil, and the transducer. The units communicate via an inductive connection through the intact skin [128], [129].

**Advantages:** MRI compatible to 1.5 T.

**Disadvantages:** Big audio-processor, high contact pressure.

**Indications:** Adults with uni- or bilateral conductive hearing loss with a difference between air and bone conduction of an average of at least 20 dB and normal or nearly normal sensorineural hearing (PTABC >30 dB HL) [129].

**Surgical technique:** Surgery can be performed under local or general anesthesia. After retroauricular incision, a bone bed is created for insertion of the implant which is fixed with titanium screws. The intervention can be compared to bonebridge surgery [130]. 

**Results: **The output signal of the BCI is sufficient for skin thicknesses of 2–8 mm, the distortion in the speech frequencies is <8% for an input sound pressure level of 70 dB. In the first patients, the functional hearing gain with BCI compared to the untreated situation was 31±8.0 dB; speech recognition improved by 51.2±8.9%. Also the subjective perception of the patients regarding the quality of life (measured with the APHAB and GBI) showed a statistically significant improvement [129].

**Discussion:** The BCI outcome in the above-mentioned study was better than or equivalent to the Ponto Pro Power (Oticon Company) that was used for one month before BCI implantation [129].

##### 4.2.2.2 Med-EL Bonebridge

**Figure 18:**
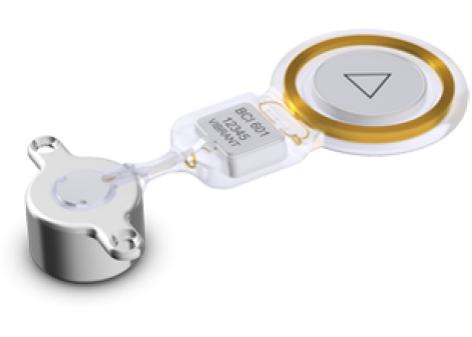
Structure of the Med-EL Bonebridge An animated presentation is found on https://www.youtube.com/watch?v=R1EJkCoXlrI.

The Bonebridge (Med-EL Company, Figure 18 (Fig. 18)) is an active partially implantable bone conduction system. An electromagnetic transducer (bone conduction floating mass transducer, BC-FMT) is fixed at the skull bone and transmits actively sound waves via the bone to the inner ear. The external unit with 2 microphones, speech processor, and battery transmits the acoustic signal transcutaneously with an amplification of up to 45 dB (at 500–4000 Hz) [99], [131].

**Advantages:** Due to the transcutaneous transmission, classical skin problems such as proliferative growth at the anchoring site and infections can be avoided. The signal is independent from the thickness of the skin and hair. It is MRI compatible up to 1.5 T.

**Disadvantages:** The implant is rather big, which makes the surgical procedure difficult. Exposition of the sinus and/or dura often cannot be avoided.

**Indications:** The Bonebridge is indicated in cases of conductive hearing loss, combined hearing impairment, or unilateral sensorineural deafness in adults and children from the age of 5 years. In the context of conductive and combined hearing loss, the bone conduction threshold should not be lower than 45 dB; for unilateral deafness, the air conduction threshold should not be below 20 dB in the better hearing contralateral ear [131].

**Surgical technique: **Surgery can be performed under local or general anesthesia. A previous CT-assisted simulation supports the intervention since the size of the implant may cause problems especially in previously operative patients with a radical cavity.

**Figure 19:**
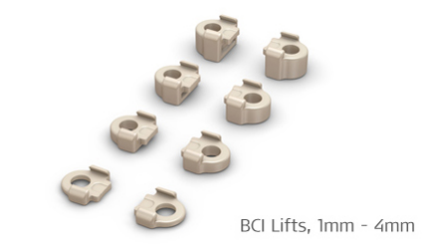
Lifter system for lifting the skin in the context of Bonebridge

After identification of the correct position, the bone bed is created and the implant is fixed with titanium screws. Meanwhile, BCI lifts as “washer” are available that facilitate the insertion of the implant (Figure 19 (Fig. 19)). So it is no longer obligatory to completely expose the dura or the sinus [99].

**Results:** In a first study with 12 adults, the functional hearing improvement after 3 months amounted to about 25 dB [132], later studies reached up to 43 dB [131], [133], [134], [135]. The speech recognition improved in quiet by 80.0±13.8% and in noise by 45.8±14.0% [136]. The authors of a study from 2014 compared the preoperative bone conduction of 23 patients with the improvement due to the implant and came to the conclusion that the preoperative threshold should not be worse than 45 dB HL. So they confirm the recommendations of the manufacturer [137].

Intraoperatively and postoperatively no particular complications were observed, over all studies only 1 of 165 patients needed revision surgery.

**Discussion: **The Bonebridge has many components in common with the Soundbridge (coil, electronic demodulator) that is in use since 1997. So a comparable compatibility was expected in the long term. Actually, the Bonebridge had a significantly lower complication rate after a follow-up of 1 year [138], [139]. Regarding the lifters that are available in the system for lifting the skin, no data could be collected up to now.

The functional results of Baha and Bonebridge are similar; for the Baha, the amplification of the low frequencies was higher, for the Bonebridge the high frequencies were higher. To improve the significance of those studies, data should be collected over a longer follow-up time.

## 5 Conclusion and outlook

Formerly, the mere sensory hearing loss was the only indication for fully or partially implantable hearing systems. Due to the rapid development of conventional hearing aids, especially the combined hearing loss turned out to be the most relevant indication in the last years. Implantable hearing systems proved their advantage over conventional hearing aids and contributed to a significantly increased quality of life.

Depending on the bone conduction threshold, different options are available. Patients with low-grade sensorineural hearing loss generally benefit from transcutaneous BCI while percutaneous BCI systems are more appropriate in the transition to moderate hearing impairment. For combined hearing losses with significant moderate or high-grade cochlear hearing loss, the provision of an AMEI (e.g. Soundbridge Codacs) might be suitable.

Despite all progress of the last years in the context of implantable hearing systems, many patients still feel insecure. In comparison to conventional hearing aids, the functional hearing and speech discrimination are only slightly better so that the main application is performed as secondary treatment of patients with incompatibilities or surgeries of the middle ear. Here, implants are a valuable and confirmed addition to the therapeutic inventory.

Because of the different fields of application and study designs, the data on the single systems are difficult to compare. For nearly every commercially available product also reports on failure and undesired side effects are found beside convincing outcomes. Further long-term studies are essential to make reliable statements which patients may benefit from which system in the long term.

## Notes

### Competing interests

The author received lecture fees and travel expenses from the company MED-EL in the past.

## Figures and Tables

**Table 1:**
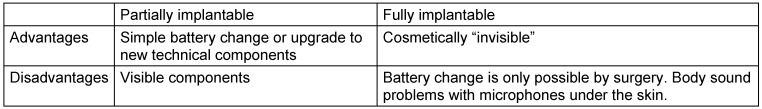
Advantages and disadvantages of fully and partially implantable hearing systems (based on [8])

**Table 2:**
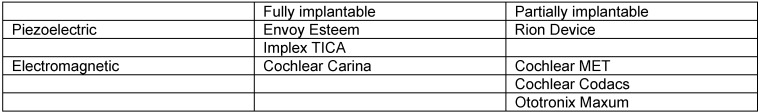
Overview of AMEI

**Table 3:**
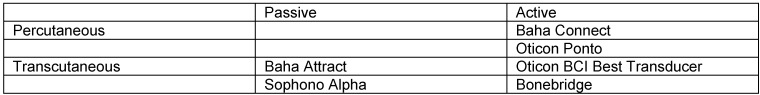
Overview of BCI
